# Probiotic Interventions Alleviate Food Allergy Symptoms Correlated With Cesarean Section: A Murine Model

**DOI:** 10.3389/fimmu.2021.741371

**Published:** 2021-09-28

**Authors:** Bi-Ying Jin, Zhen Li, Ya-Nan Xia, Li-Xiang Li, Zi-Xiao Zhao, Xiao-Yu Li, Yan Li, Bing Li, Ru-Chen Zhou, Shi-Chen Fu, Shi-Yang Li, Yan-Qing Li

**Affiliations:** ^1^ Department of Gastroenterology, Qilu Hospital of Shandong University, Jinan, China; ^2^ Laboratory of Translational Gastroenterology, Qilu Hospital of Shandong University, Jinan, China; ^3^ Robot Engineering Laboratory for Precise Diagnosis and Therapy of Gastrointestinal Tumor, Qilu Hospital of Shandong University, Jinan, China; ^4^ Advanced Medical Research Institute, Cheeloo College of Medicine, Shandong University, Jinan, China

**Keywords:** food allergy, cesarean section, intestinal microbiota, probiotics, Th2 response, tight junction

## Abstract

Delivery by cesarean section (CS) is linked to an increased incidence of food allergies in children and affects early gut microbiota colonization. Furthermore, emerging evidence has connected disordered intestinal microbiota to food allergies. Here, we investigated the impact of CS on a rat model for food allergy to ovalbumin (OVA). Rats delivered by CS were found to be more responsive to OVA sensitization than vaginally born ones, displaying a greater reduction in rectal temperature upon challenge, worse diarrhea, and higher levels of OVA-specific antibodies and histamine. 16S rRNA sequencing of feces revealed reduced levels of *Lactobacillus* and *Bifidobacterium* in the CS rats. Preventative supplementation with a probiotic combination containing *Lactobacillus* and *Bifidobacterium* could protect CS rats against an allergic response to OVA, indicating that the microbiota dysbiosis contributes to CS-related response. Additionally, probiotic intervention early in life might help to rebuild aberrant Th2 responses and tight junction proteins, both of which have been linked to CS-related high allergic reactions. Taken together, this study shows that disordered intestinal microbiota plays an essential role in the pathogenesis of food allergy mediated by CS. More importantly, interventions that modulate the microbiota composition in early life are therapeutically relevant for CS-related food allergies.

## Introduction

Recent decades have witnessed an increasing prevalence of delivery by cesarean section (CS) across the globe (1). Furthermore, a growing body of work has implicated CS in food allergy (2, 3) and several other immune-mediated diseases, including asthma, inflammatory bowel disease, eczema, atopic dermatitis, and immune deficiencies (4–7). CS in mice has also been shown to result in marked and long-term behavioral changes, regulatory immunity shifts, and increased sensitivity to oxazolone-induced colitis compared with mice delivered vaginally in offspring (8–11).

Food allergies are triggered by normally innocuous dietary antigens, and children with these allergies face food and social restrictions, which may lead to impaired quality of life (12). Despite the potentially life-threatening nature of food allergies, their precise pathogenesis remains unclear. Several epidemiological studies have shown that the composition of gut microbiota differs between allergic and healthy children (13–15). The microbial flora appears to have a significant impact on programming oral tolerance, and the development of Th2 and IgE responses to food antigens is linked to the absence of microbiota in germ-free mice (16, 17). The critical role of intestinal bacteria in food allergy has been further demonstrated in murine models. For example, fecal transfer from infants with cow’s milk allergy and from mice sensitized by ovalbumin (OVA) induces food allergies in the recipient germ-free mice (18, 19). Nevertheless, germ-free animals are specialized models, and it is uncertain if more medically relevant changes in the microbiota makeup early in life could have long-term immunological and anaphylactic effects.

The composition of the gut microbiota begins to develop mainly after birth and is infected by several factors, including mode of delivery (20), breastfeeding (21), antibiotics (22), and environmental exposure (23, 24), all of which can have enduring health consequences for the offspring. CS delivery leads to a distinct pattern of microbiota colonization in newborns, exhibiting a lower relative abundance of vaginal bacteria and a higher level of opportunistic pathogens associated with the hospital environment (20, 25). The hygiene hypothesis suggests that reduced exposure to microbial stimuli in early life programs the immune system toward a Th2-type allergic response (16, 26). Although an important window of opportunity for immune education has been recently demonstrated (27), CS-induced changes in the gut microbiota have been largely neglected in the clinical and preclinical research on food allergy.

Compared to infants born by vaginal delivery (VD), those delivered by CS are characterized by a lower abundance of *Lactobacillus*, *Prevotella*, and *Bifidobacterium* (20, 28, 29). Therefore, restoring the gut microbiota of CS-delivered babies by maternal vaginal fluid exposure or the inclusion of probiotics into formula may reduce disease risks in adolescence and even into adulthood (30, 31). *Bifidobacterium* is the neonate’s early gut colonizer within the first days and weeks after birth (32). *Bifidobacterium longum* subsp. *infantis* (used to be called *Bifidobacterium infantis*), in particular, dominates the gut microbiota of breastfed infants and helps the host limiting excessive inflammation, improving intestinal barrier function, and increasing acetate production (33). And infants supplied with *Bifidobacterium longum* subsp. *infantis* have shown an improvement in the symptoms of allergic rhinitis, asthma, and diarrhea in clinical trials (34, 35). *Lactobacillus* strains are important components of the human and animal microbiomes and are among the most commonly used probiotics (36). Early *Lactobacillus rhamnosus GG* supplementation helps to prevent eczema and asthma (37), and orally administered *Lactobacillus acidophilus* significantly ameliorates the symptoms of atopic dermatitis in children (38). Beneficial effects regarding the development of allergic diseases have also been suggested to come through supplementation of other probiotics. Intragastrical administration of *Enterococcus faecalis* hampers the establishment of the OVA-induced allergic immune response (39). *Bacillus cereus* has been shown to regulate immunological responses (40), and treatment with its germination lipoprotein to protect mice against sensitization and airway inflammation in an asthma model (41).

In this work, we employed an OVA-induced allergic model to investigate the impact of CS on experimental food allergy in rats and gut-microbiota-targeted treatments in reversing such effects. We supplied pups with a probiotic mixture from birth to see whether it could avert the negative consequences associated with CS. This study shows a relationship between alterations in the microbiota and the severity of food allergy from a perspective of birth mode.

## Materials and Methods

### Animals and CS

Pregnant Sprague Dawley rats were purchased from Beijing SPF Biotechnology Co., Ltd, and kept in the Experimental Animal Center of Shandong University. All animals were maintained in pathogen-free environments (22 ± 2°C, 12-h light-dark cycle) with *ad libitum* access to food and water. All experiments performed on animals were approved by the Ethics Committee on animal experiment of Qilu Hospital of Shandong University (DWLL-2020-07).

On day 20 of pregnancy, CS was performed by hysterectomy and an incision was made. The pups were gently placed on sterile gauze with a heating pad underneath. Each pup was massaged with sterile cotton swabs until spontaneous breathing occurred, and then the umbilical cord was cut. The pups were transferred to a foster mother that gave birth on the same day within 30 min. Besides, the pups born naturally were raised by their biological mother, and these litters served as VD controls. Offspring were weaned on the 21st day after birth and group-housed with three rats per cage. They were then numbered, and there were corresponding records on the label outside the cage. In each group, two litters were utilized, and no litter differences were found.

### OVA-Induced Food Allergy

On Day 21 the healthy male VD and CS rats were randomly divided into the sensitization group and the control group, respectively. The sensitization group was administrated 1 mg OVA (Sigma-Aldrich, St. Louis, MO, USA) dissolved in 1 ml phosphate-buffered saline (PBS), orally every day for 49 days, while the control group was administrated 1 ml PBS instead. Animals were excluded if anorexia, rapid weight loss, or mental and behavioral burnout occurred after sensitization. And a humane endpoint of animal euthanasia would be carried out. On Day 70, all the rats were orally challenged with 100 mg OVA in 1 ml PBS. Anaphylaxis was assessed by measuring changes in rectal temperature 25 min after challenge by a digital thermometer. To evaluate the severity of diarrhea, stool condition was recorded as follows: solid (score 0), funicular (score 1), slurry (score 2), and watery (score 3). Then blood samples were collected from the caudal vein. All rats were sacrificed 8 h after challenge (42), and ileal tissue samples were collected for further study. The total number of rats in each group were as follows: VD_PBS (n = 9), VD_OVA (n = 8), CS_PBS (n = 8), CS_OVA (n = 8).

### Probiotic Administration

After birth, the pups delivered by CS received mixed probiotics Siliankang (Hangzhou Grand Pharmaceutical, Ltd., China) in PBS (the CSP group, CSP = cesarean section with probiotic intervention) or PBS (the CSO group, CSO = the cesarean section offspring) daily for 21 days by gavage, which has been approved by the National Medical Products Administration in China. The mixture given regularly containing 1.86×10^6^ CFU *Bifidobacterium longum* subsp. *infantis* (*Bifidobacterium infantis*) CGMCC 0460.1, 1.86×10^6^ CFU *Lactobacillus acidophilus* CGMCC 0460.2, 1.86×10^5^ CFU *Enterococcus faecalis* CGMCC 0460.3, and 1.86×10^4^ CFU *Bacillus cereus* CGMCC 0460.4 (calculated from the equivalent dosage for neonatal rats). On Day 21, male CSO and CSP rats were subjected to later OVA intervention. The total number of rats in each group: CSO (n = 7), CSP (n = 7).

### Serum OVA-Specific IgE and IgG Analyzing

Blood was collected from the rats’ tail veins. After centrifugation, the sera were collected and stored at −80°C until analyzed. The levels of OVA-specific IgE and IgG were measured by enzyme-linked immunosorbent assays (ELISA). In brief, Nunc™ MaxiSorp™ ELISA plates (BioLegend, San Diego, USA) were coated with 10 μg/ml OVA in coating buffer (BioLegend, San Diego, USA) overnight for OVA-specific IgE detection, 1 μg/ml for OVA-specific IgG detection. Serum samples (1/10 dilutions in Assay Diluent B, BioLegend) were added to the plates and incubated overnight at 4°C. Biotin-conjugated mouse anti-Rat IgE secondary antibody (1:2,000; Thermo Fisher Scientific, USA) or biotin-conjugated mouse anti-Rat IgG (H+L) secondary antibody (1:100,000; Thermo Fisher Scientific, USA) was separately added to the plates. Thereafter HRP avidin (BioLegend, San Diego, USA), diluted 1:2,000, was added. Plates were developed with tetramethylbenzidine (TMB) for 15 min at room temperature, stopped by the addition of Stop Solution (BioLegend), and read at 450 and 570 nm. The antibody levels were revealed from the observed magnitude difference between OD450 and OD570. Values less than 0.1 were regarded as undetectable and excluded in this assay.

### Histamine and Cytokines Measurement *via* ELISA

The total protein of ileal tissues was extracted by hypothermic ultrasonication and quantified by BCA assay (BCA Protein Assay kit, ABP Biosciences, USA). Histamine in tissue and serum was measured by a Rat histamine ELISA kit (Anoric Biotechnology, CO., Ltd, Tianjin, China). The histamine content in the tissue was normalized by dividing the total protein concentration of each sample (ng/g protein). IL-4 and IL-10 were evaluated using Rat IL-4 high sensitivity ELISA kit (Multi Sciences Biotech, CO., Ltd.) and Rat IL-10 high sensitivity ELISA kit (Multi Sciences Biotech, CO., Ltd.) and normalized in the same manner (ng/g protein). Both BCA and ELISA assays were performed according to the manufacturer’s instructions. OD values less than 0.1 were regarded as undetectable and excluded in this assay.

### Western Blot

The total proteins of tissues were obtained using RIPA Lysis Buffer (Solarbio Life Science, Beijing, China) and quantified. Thereafter, protein was denatured and separated by 10% sodium dodecyl sulfate-polyacrylamide gel electrophoresis (SDS-PAGE) and transferred to a polyvinylidene difluoride (PVDF) membrane. The membrane was treated overnight at 4°C with primary antibody before being incubated for 60 min at 22°C with the secondary antibody. The bands were detected using an improved chemiluminescent substrate (Millipore, USA). Antibodies: anti-β-actin antibody (1:10,000; Proteintech, Hubei, China), ZO-1 Polyclonal Antibody (1:500; Thermo Fisher Scientific, USA, 61-7300), Occludin Polyclonal Antibody (1:200; Thermo Fisher Scientific, USA, 71-1500), Claudin-1 Polyclonal Antibody (1:250; Thermo Fisher Scientific, USA, 51-9000), goat anti-mouse IgG (1:1,000; Zhongshan GoldBridge, Beijing, China), and goat anti-rabbit IgG (1:1,000; Zhongshan Gold Bridge, Beijing, China).

### Immunohistochemistry (IHC) and Hematoxylin-Eosin (HE) Staining

The ileal tissues of rats were fixed overnight at 4°C in 4% paraformaldehyde and were subsequently embedded in paraffin. Fixed and embedded tissues were sliced into 4 μm slides. IHC staining was performed using Zo-1 Polyclonal Antibody (1:2,000), Occludin Polyclonal Antibody (1:1,000), and Claudin-1 Polyclonal Antibody (1:1,000). Sections of paraffin-embedded tissues were subjected to HE staining to measure the severity of inflammation.

### RNA Extraction and Quantitative Real-Time PCR

Total RNA was extracted from the middle part of ileum using TRIzol reagent (Invitrogen, CA, USA) according to the manufacturer’s instructions. The ReverTra Ace qPCR RT Kit (Toyobo, Osaka, Japan) was then used to make cDNA. On an Applied Biosystems StepOne Real-Time PCR System (Thermo, Waltham, MA, USA), all reactions were performed in triplicate. β-Actin was utilized as an endogenous control. The primer sequences used for real-time PCR analysis are as follows: β-Actin (forward, 5’-CTCTGTGTGGATTGGTGGCT-3’, reverse, 5’-CGCAGCTCAGTAACAGTCCG-3’), IL-4 (forward, 5’-TGTAGAGGTGTCAGCGGTCT-3’, reverse, 5’-TCAGTGTTGTGAGCGTGGAC-3’), IL-10 (forward, 5’-TAACTGCACCCACTTCCCAG-3’, reverse, 5’-TGGCAACCCAAGTAACCCTTAAA-3’).

### 16S Ribosomal RNA Gene Sequencing and Microbiota Analysis

On Day 7, eight rats in the VD and CS groups were sacrificed for their intestinal content. On Day 21 and 70, feces were collected. Rat feces samples were rapidly frozen in liquid nitrogen and kept at −80°C. The FastDNA SPIN Kit (MP Biomedicals, Irvine, CA, USA) was used to extract microbial DNA from fecal samples according to kit protocols. The V3-V4 regions of the bacteria 16S rRNA gene were amplified by PCR using the primers (338F: ACTCCTACGGGAGGCAGCA, 806R: GGACTACHVGGGTWTCTAAT). The amplicons were subsequently purified by gel extraction (AxyPrep DNA Gel Extraction Kit, Axygen, Union City, CA, USA) and quantified by QuantiFluor™-ST (Promega) according to protocols. The purified DNA amplicons were then sequenced by Majorbio (Shanghai, China) on Illumina MiSeq platform.

Harvested reads were mostly analyzed on Majorbio Cloud Platform (https://cloud.majorbio.com/). Samples with <30,000 reads were excluded from the analysis. All estimates were calculated by evenly sampling at each time point to normalize the differences in sequencing depth. Each sample’s microbial richness was assessed using the Ace and Chao indexes, as well as the Shannon index for alpha diversity. PCoA on operational taxonomy units (OTU) level was performed based on binary-jaccard distances. The structural differences between VD and CS rats were investigated using ANOSIM and 999 times permutation testing. The relative abundance of the genera was evaluated using two-sided Student’s t-test to compare between groups. The significantly discriminant taxa in each group were identified by the linear discriminant analysis effect size (LEfSe) analysis from phylum to genus level (using one-against-all comparisons) with the value of the Kruskal–Wallis rank-sum test set to 0.05. Significant taxa were used to generate taxonomic cladograms illustrating differences among different groups and visualized on Galaxy web application (http://huttenhower.org/galaxy/). Spearman correlation coefficients were calculated for relationships between interleukin levels and taxonomic abundances of the 30 most predominant genera and shown as correlation heatmap (correlation edges with spearman’s coefficient > 0.75/< −0.75).

### Data Analysis

Results were analyzed on GraphPad Prism 5.0c (GraphPad Software, La Jolla, CA, USA). Differences between multiple groups were evaluated using one-way ANOVA. Student’s t-test or one-way ANOVA was applied to compare two groups. Data were expressed as means ± SEM in the column plot. Differences were considered significant at P < 0.05. Kruskal–Wallis non-parametric test was used to assess different taxa among the groups. Correlations between the two parameters were assessed by Spearman rank correlation.

## Results

### CS Increases Disease Severity in OVA-Induced Food Allergy

**Figure 1:**
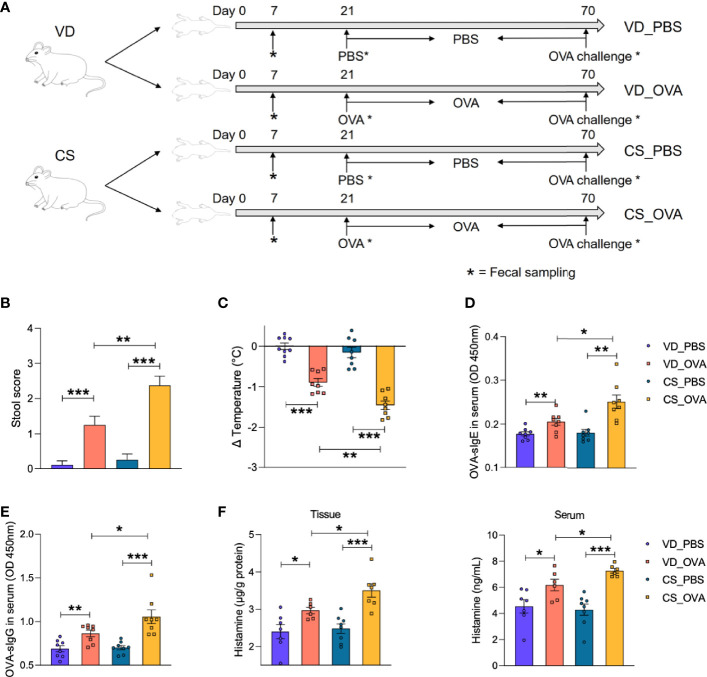
CS increases disease severity in OVA-induced food allergy. **(A)** Schematic diagram of VD and CS rats submitted to food allergy. **(B)** The severity of allergic diarrhea was judged by stool score. (VD_PBS n = 9, VD_OVA n = 8, CS_PBS n = 8, CS_OVA n = 8). **(C)** Changes in rectal temperature at 25 min after challenge (VD_PBS n = 9, VD_OVA n = 8, CS_PBS n = 8, CS_OVA n = 8). **(D, E)** OD 450 was used to indicate the relative OVA-specific IgE **(D)** and IgG **(E)** levels in the serum. n = 8. **(F)** Histamine level in the ileum or serum measured by ELISA. n ≥ 6. For **(B)**, bars represent mean and SEM. For **(C–F)**, circles and squares represent individual rats, and bars represent mean ± SEM. Statistical significance was determined by two-sided Student’s t-test or one way ANOVA, *P < 0.05, **P < 0.01, ***P < 0.001. VD, vaginal delivery; CS, cesarean section.

To confirm if delivery mode has an effect on food allergy, we developed a rat food allergy model based on CS (see detailed description in the *Materials and Methods* section). Sprague Dawley rats, delivered by CS or vaginally were sensitized daily with 1 mg OVA (dissolved in 1 ml PBS) or 1 ml PBS alone, initiating on Day 21 and ending on Day 69. All rats in the four groups were orally challenged with 100 mg OVA on Day 70 and sacrificed 8 h later for further analysis (**Figure 1A**).

To evaluate our food allergy model, we first assessed the allergic symptoms based on fecal score and rectal temperature change. Marked diarrhea and hypothermia in the OVA-sensitized groups revealed the success of the food allergy model implementation (**Figures 1B, C**). Moreover, compared to the VD_OVA group, CS_OVA rats had considerably worse diarrhea and larger temperature variations. Consistent with the allergic symptoms, serum OVA-specific IgE and IgG levels were significantly elevated in the sensitized groups and amplified by CS, which provided solid serological evidence for the food allergy model (**Figures 1D, E**). Levels of the allergy marker histamine were significantly higher in both tissue and serum of OVA-treated CS rats than those in VD rats, indicating more severe anaphylaxis in CS rats (**Figure 1F**). However, HE staining of the ileum after challenge revealed no pathological changes (see also **Figure S1**). Together, these findings imply that CS worsens the severity of illness in rats with an OVA-induced food allergy.

### CS Dysregulates the Expression of Tight Junction Proteins and Upregulates the Levels of Th2 Cytokines

**Figure 2:**
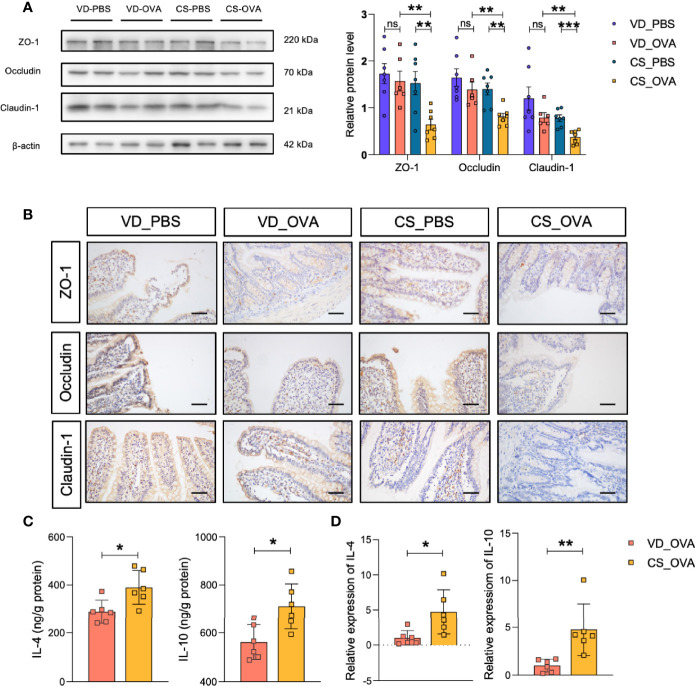
CS dysregulates the expression of tight junction proteins and upregulates the levels of Th2 cytokines. **(A)** Western blot analysis of the tight junction proteins ZO-1, occludin, and claudin-1 in the ileum of four groups. β-actin was used as the internal control (left panel). Grayscale statistics of tight junction proteins relative to β-actin (Right panel). n ≥ 6. **(B)** Representative images of immunohistochemistry staining for ZO-1, occludin, and claudin-1 proteins. Scale bar = 50 μm. **(C, D)** IL-4 and IL-10 levels measured by ELISA **(C)** and q-RT-PCR **(D)**. n = 6. Data are expressed as means ± SEM. Statistical significance was determined by two-sided Student’s t-test or one way ANOVA, *P < 0.05, **P < 0.01, ***P < 0.001; ns, no significance; VD, vaginal delivery; CS, cesarean section.

We hypothesized that the intestinal barrier of rats subjected to OVA was compromised because of their diarrhea symptoms. Therefore, we analyzed the expression of tight junction proteins, which are the most adhesive junctional complexes in the epithelium, maintain the intestinal barrier, and regulate its permeability (43). Western blot analysis of ileal proteins from OVA-treated rats revealed that the CS community had lower expression levels of ZO-1, occludin, and claudin-1 than those in the VD group (**Figure 2A**). Surprisingly, OVA sensitization had practically little effect on the tight junction proteins of VD rats. Furthermore, ileal tissues from VD and CS rats were subjected to IHC staining to verify our conclusion; compared with VD_OVA animals, the expression levels of ZO-1, occludin, and claudin-1 were lower in CS_OVA animals (**Figure 2B**). These data indicate that when exposed to dietary antigens, the intestinal barrier becomes more impaired in rats delivered by CS than VD.

Food allergy is associated with the dysregulation of Th2 cytokines, such as IL-4 and IL-10, suggesting that the Th2 response is critical in food allergy (44, 45). Thus, we investigated the expression of the Th2 markers in the ileum. ELISA indicated that the CS_OVA group had higher levels of both IL-4 and IL-10 than those of the VD_OVA group (**Figure 2C**). The qRT-PCR results were nearly identical to those of the ELISA (**Figure 2D**). These findings demonstrate that the levels of Th2 cytokines, which are involved in food allergy, are higher in the CS group.

### Food Allergy Is Strongly Associated With Changes in Gut Microbiota Caused by CS

**Figure 3:**
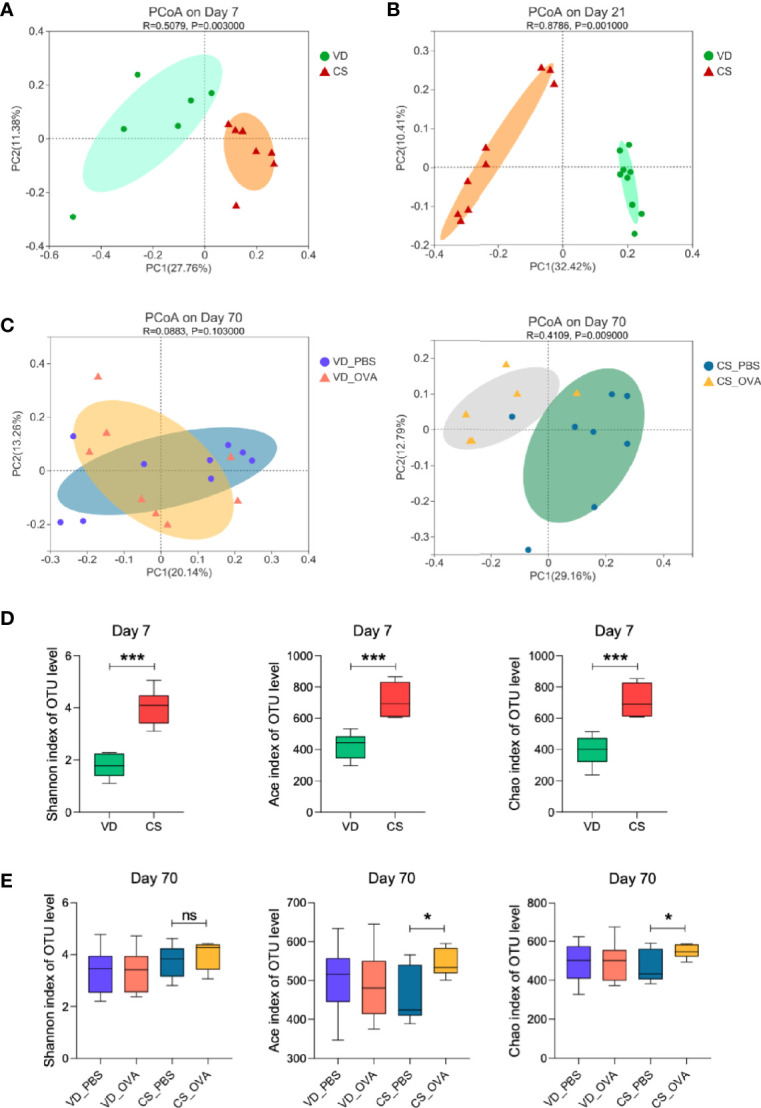
Mode of delivery affects the composition of the microbiota. **(A–C)** PCoA of fecal microbiota composition in VD and CS rats on Days 7 **(A)**, 21 **(B)**, and 70 **(C)**. PCoA is based on binary-jaccard distance. ANOSIM and 999 times permutation tests were utilized. **(D, E)** Shannon, Ace, and Chao indexes on Days 7 **(D)** and 70 **(E)**. Data are expressed as means ± SEM, and statistical significance was determined by two-sided Student’s t-test or one way ANOVA. *P < 0.05, ***P < 0.001; ns, no significance. **(A, D)** Day 7 (VD n = 6, CS n =7). **(B)** Day 21 (VD n = 9, CS n =9). **(C, E)** Day 70 (VD_PBS n = 9, VD_OVA n = 8, CS_PBS n = 8, CS_OVA n = 6). VD, vaginal delivery; CS, cesarean section.

We postulated that variations in intestinal microbiota between CS and VD groups were the underlying cause of disparities in clinical features, intestinal barrier permeability, and immunological responses. Therefore, 16S rRNA gene sequencing was performed to profile the gut microbiota composition of the CS and VD groups on Days 7, 21, and 70. PCoA on OTU level showed that the structure of the microbial community significantly differed in the CS offspring on Days 7 (*p* = 0.003) and 21 (*p* = 0.001) compared with VD (**Figures 3A, B**). We also detected a significant difference (*p* = 0.009) in microbiome composition between the sensitized and control groups among CS rats, but no comparable change was observed for the VD rats (*p* = 0.103), which implies that CS magnified the alteration in the microbial community between PBS- and OVA-treated groups (**Figure 3C**). This could also account for the differences in levels of tight junction proteins. On Day 7, Shannon index indicated a higher alpha diversity in the feces of the CS rats. Ace and Chao indices also showed that the CS group had more microbial diversity than the VD group (**Figure 3D**). At the end of sensitization (Day 70), bacterial richness and diversity based on these three indices did not differ significantly between VD_PBS and VD_OVA groups. Despite no difference in Shannon index, the CS_OVA group had higher Ace and Chao indices than the CS_PBS group (**Figure 3E**). These data reveal that changes in microbiota composition caused by CS persist in rats with OVA food allergy.

**Figure 4:**
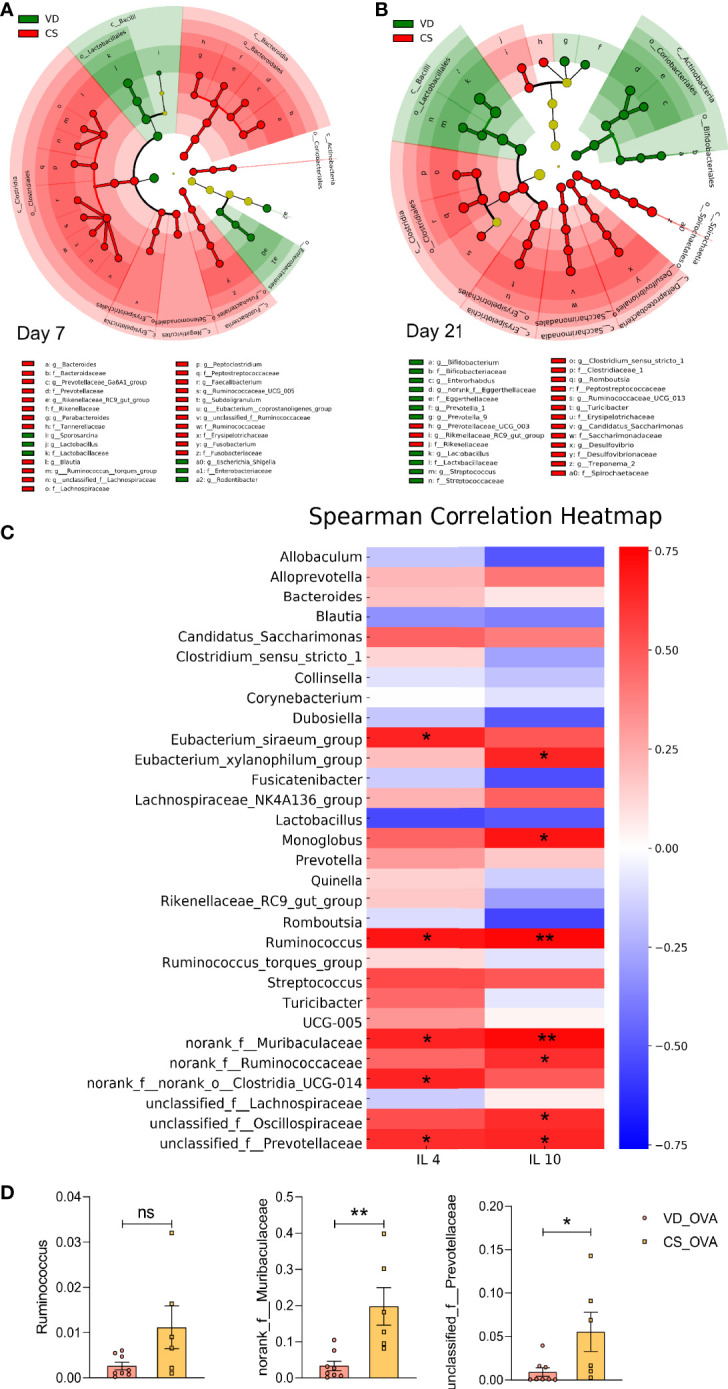
Food allergy is linked to the differences in microbiota induced by CS. **(A, B)** Cladogram based on LEfSe analysis from phylum to genus level with the value of the Kruskal–Wallis rank-sum test set to 0.05 on Days 7 **(A)** and 21 **(B)**. **(A)** Day 7 (VD n = 6, CS n =7). **(B)** Day 21 (VD n = 9, CS n =9). **(C)** Spearman Correlation heatmap indicated the correlation between the protein levels of IL-4 and IL-10 and abundance of genera (n = 6). Positive and negative correlations are shown in red and blue blocks, respectively. Spearman’s correlation coefficients >0.75/< −0.75. **(D)** The relative abundance of some specific microbes that are positively correlated with the levels of IL-4 and IL-10 in **(C)** on Day 70 (VD_OVA n = 8, CS_OVA n = 6). *P < 0.05, **P < 0.01; ns, no significance; VD, vaginal delivery; CS, cesarean section.

LDA was used to identify the most differentially abundant taxa, and cladograms were generated. Using the LEfSe method, *Lactobacillus* sequences were found to be most reduced in the CS rats on Day 7 (LDA value = 5.36) and Day 21 (LDA value = 5.06), while sequences from the *Bifidobacterium* genus were also substantially lowered (LDA value = 3.68) on Day 21. (**Figures 4A, B**; see also **Figure S2** and **Figure S3**). We discovered several other significant taxa that have been noted in previous publications, indicating that a change in gut bacteria is a fundamental difference between children with and without food allergies (13–15). CS subjects had higher levels of *Faecalibacterium*, *Clostridium sensu stricto*, and *Subdoligranulum* genera, which are increased in food-allergic children compared to healthy children. In contrast, *Lactobacillus*, *Bifidobacterium*, *Streptococcus*, and *Prevotella*, which have been reported to be negatively related to food allergy, were enriched in VD controls.

According to our data, the CS rat population was shown to produce more Th2 cytokines. To determine whether there was a link between greater levels of Th2 cytokines and microbiota dysbiosis, we examined the relationship between interleukins and taxonomic abundances following OVA sensitization. The heatmap indicated that levels of the dysregulated bacteria, such as *Ruminococcus*, *norank_f: Muribaculaceae*, and *unclassified_f:Prevotellaceae*, were positively correlated with the protein levels of IL-4 and IL-10 (**Figure 4C**). The CS_OVA group showed a higher relative abundance of *Ruminococcus* (*p* = 0.0619), *norank_f: Muribaculaceae* (*p* = 0.0043), and *unclassified_f:Prevotellaceae* (*p* = 0.0416) than the VD_OVA group (**Figure 4D**). Thus, we speculated that these bacteria are involved in the development of food allergy. *Ruminococcus*, in particular, is a notorious bacterium in food allergy cases (13). Collectively, these findings suggest that the CS offspring were more likely to develop food allergy due to changes in their gut microbiota. For the first time, we demonstrated that higher levels of Th2 cytokines, triggered by microbiota differences, play a significant role in the CS-mediated food allergy model in rats.

### Probiotic Intervention Modulates Intestinal Microbiota Structure of Rats Delivered by CS

**Figure 5:**
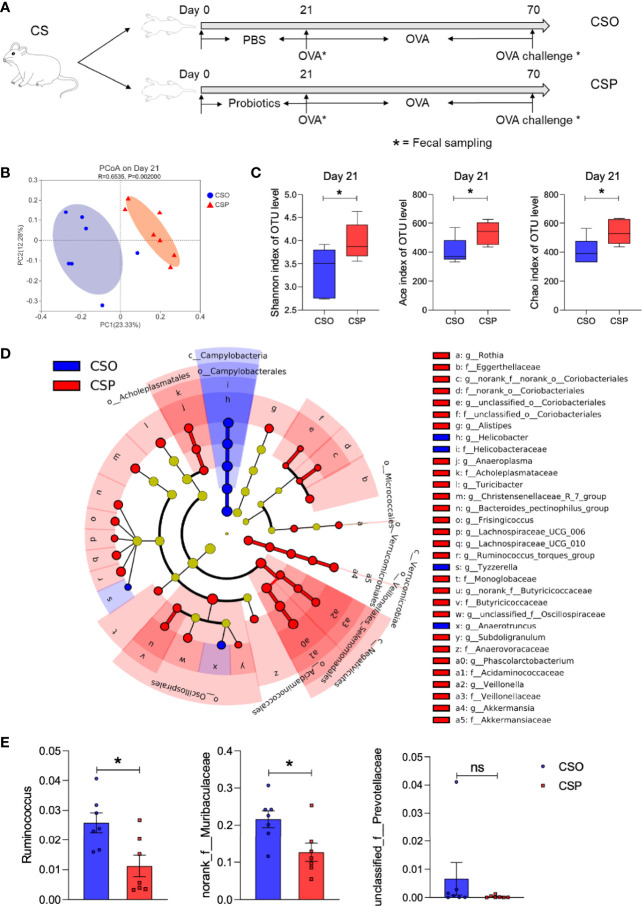
Probiotic intervention modulates intestinal microbiota structure of CS rats. **(A)** Schematic diagram of CS rats submitted to probiotics intervention and food allergy. **(B)** PCoA of fecal microbiota composition in CSO and CSP rats on Day 21. n = 7. ANOSIM and 999 times permutation tests were utilized. **(C)** Shannon, Ace, and Chao indexes on Day 21. n = 7. **(D)** Cladogram of CS and CSP based on LEfSe analysis from phylum to genus level with the value of the Kruskal–Wallis rank-sum test set to 0.05. n = 7. **(E)** The relative abundance of some specific microbes on Day 70. n = 7. Data are expressed as means ± SEM, statistical significance was determined by two-sided Student’s t-test or one way ANOVA, *P < 0.05; ns, no significance; CSO, the cesarean section offspring; CSP, cesarean section with probiotic intervention.

Since *Lactobacillus* and *Bifidobacterium* were found to be lower in CS rats before weaning, and considering the critical role of intestinal microbiota in regulating allergic responses, we hypothesized that *Lactobacillus* and *Bifidobacterium* supplementation to CS rats in early life might reverse the food allergy symptoms induced by OVA sensitization. Based on this assumption, we first examined whether supplying a widely available probiotic tablet containing *Lactobacillus acidophilus*, *Bifidobacterium longum* subsp. *infantis*, *Enterococcus faecalis*, and *Bacillus cereus* from birth until Day 21 could rescue the CS-related intestinal microbiota disorder (**Figure 5A**). PCoA on OTU level showed that the structure of the intestinal microbial community was considerably altered following probiotic administration for 21 days (**Figure 5B**). On Day 21, the CSP group had a higher alpha diversity and microbial richness than the CSO group, according to the Shannon, Ace, and Chao indices (**Figure 5C**). The cladogram revealed that the CSP group was enriched in *Alistipes* and *Veillonella* genus (**Figure 5D**); wherein both genera are negatively related to food allergy (13). Except for *unclassified_f:Prevotellaceae*, the relative abundance of the genera positively correlated with Th2 cytokines (*Ruminococcus* and *norank_f: Muribaculaceae*; **Figure 4C**) was considerably lower in the CSP group than in the CSO group on Day 70 (**Figure 5E**). These findings indicate that probiotic intervention altered the microbiota composition, inhibiting certain food allergy-related bacteria. As a result, we concluded that probiotics intervention could be an effective strategy to adjust the microbial community structure in the CS offspring.

### Probiotics Intervention After Birth Protects CS Rats Against Food Allergy

**Figure 6:**
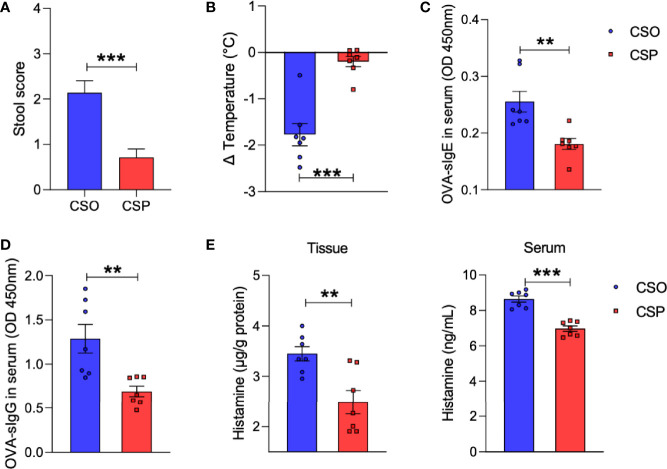
Probiotics reduce allergic response to OVA. **(A)** The severity of allergic diarrhea was judged by stool score. **(B)** Changes in rectal temperature at 25 min after sensitization. **(C, D)** OD 450 was used to indicate the relative levels of OVA-specific IgE **(C)** and IgG **(D)** in serum. **(E)** Histamine level in the ileum or serum measured by ELISA. For **(A),** bars represent mean and SEM. n = 7. For **(B–E)**, circles and squares represent individual rats, and bars represent mean ± SEM. n = 7. Statistical significance was determined by two-sided Student’s t-test or one way ANOVA, **P < 0.01, ***P < 0.001. CSO, the cesarean section offspring; CSP, cesarean section with probiotic intervention.

We next explored whether probiotics given early in life can help relieve the allergy symptoms in CS rats. Probiotic administration improved diarrhea and hypothermia in the CSP group compared with the CSO group, as measured by fecal score and rectal temperature (**Figures 6A, B**). Furthermore, serological analysis demonstrated that probiotics could lower OVA-specific IgE and IgG concentrations in the serum (**Figures 6C, D**). Histamine levels decreased after probiotic gavage in both serum and tissue of sensitized CS rats (**Figure 6E**). Thus, probiotics intervention has been proven to alleviate the symptoms and immune responses of food allergy in the rat model.

**Figure 7:**
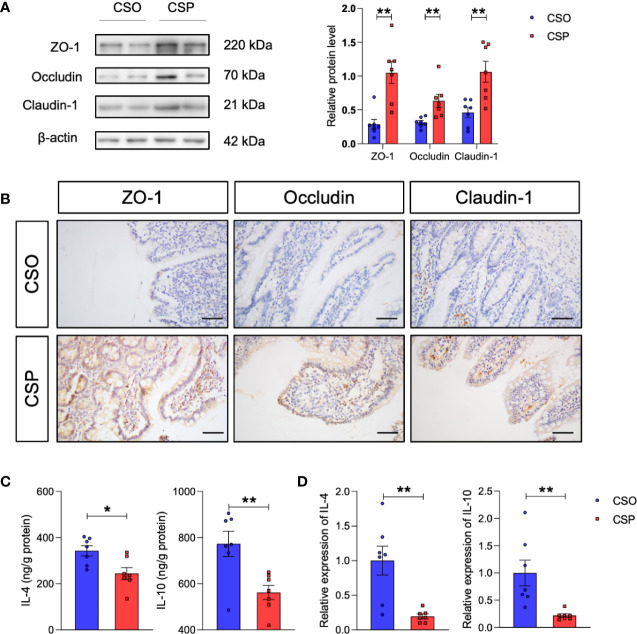
Probiotic intervention reverses the changes in tight junction proteins and Th2 cytokines induced by CS in food allergy. **(A)** Western blot analysis of the tight junction proteins ZO-1, occludin, and claudin-1 in the ileum. β-actin was used as the internal control (left panel). Grayscale statistics of tight junction proteins relative to β-actin (Right panel). n = 7. **(B)** Representative images of immunohistochemistry staining for ZO-1, occludin, and claudin-1 proteins. Scale bar = 50 μm. **(C, D)** IL-4 and IL-10 levels measured by ELISA **(C)** and q-RT-PCR **(D)**. n = 7. Data are expressed as means ± SEM. Statistical significance was determined by two-sided Student’s t-test or one way ANOVA, *P < 0.05, **P < 0.01. CSO, the cesarean section offspring; CSP, cesarean section with probiotic intervention.

Western blot analysis revealed that the levels of tight junction proteins, such as ZO-1, occludin, and claudin-1, which were damaged by OVA sensitization in CS rats, increased after probiotic intervention (**Figure 7A**). This is consistent with the IHC results (**Figure 7B**). More importantly, the Th2 response also recovered partially, which were reflected in decreased IL-4 and IL-10 levels by ELISA and qRT-PCR (**Figures 7C, D**). These results collectively suggest that *Lactobacillus* and *Bifidobacterium* supplementation is a promising strategy for preventing food allergy in the CS offspring.

## Discussion

Children born by CS are more vulnerable to food allergies during their childhood (46). The composition of the intestinal microbial community differs between healthy children and those with food allergies (13–15). Altered microbiome composition at critical stages of early life, especially during maturation of the immune system, has been implicated in later increased risks of allergic diseases (47, 48). CS results in a different colonization pattern of the gut microbiota in the neonates because they are not exposed to maternal vaginal microbiota (49). Here, we established a rat model of CS delivery to compare the severity of OVA-induced food allergy between rats delivered vaginally and by CS. Sensitized CS rats produced significantly higher serum concentrations of OVA-specific antibodies and histamine than VD rats, in addition to a greater drop in rectal temperature and worse diarrhea. This is the first time a CS-delivered animal model has been utilized to investigate food allergy, and the difference in illness severity mediated by birth modes has been shown. Previous clinical studies have demonstrated that CS significantly reduces the abundance of *Lactobacillus* and *Bifidobacterium* during infancy (20, 29). In agreement, our model displayed a significant depletion of these bacteria in the CS offspring until Day 21. In addition, *Faecalibacterium*, *Clostridium sensu stricto*, and *Subdoligranulum* were upregulated in the CS group, while the abundance of *Streptococcus* and *Prevotella* were lower compared with that of the VD group. These bacteria have also been reported to contribute significantly to food allergy (13–15). We demonstrated that CS-induced compositional alterations in the intestinal microbiota are associated with food allergies.

To explore whether supplementing *Lactobacillus* and *Bifidobacterium* at the beginning of life could improve food allergy outcomes in the CS rats, we used a widely available probiotic pill that includes these two bacteria to compensate for their loss after birth. Treatment with probiotics successfully reversed the allergic symptoms and reduced specific antibodies in the CS offspring. Thus, we found that supplementation with a mixture of *Lactobacillus acidophilus*, *Bifidobacterium longum* subsp. *infantis*, *Enterococcus faecalis*, and *Bacillus cereus* reversed the impact of birth by CS on food allergy and suggests a causal link between dysbiosis of intestinal microbiota and food allergy.

In line with our finding that early microbial intervention suppresses CS-induced food allergy, selected probiotics like *Lactobacillus rhamnosus GG* have been found to be preventive and therapeutic against food allergies (31, 50). The role of the microbiota in the occurrence of food allergy and the precise mechanism by which probiotics restore the dysbiosis of the microbial community remained largely unknown. Early maturation of a healthy gut microbiota favors a Th1 cell response (51), whereas dysbiosis of the microbiota promotes a shift in the Th1/Th2 balance toward a Th2 response (52). In this setting, later oral allergen exposure results in aberrant Th2 responses, which trigger the food allergy process, even if the allergens are harmless (53). Between sensitized VD and CS rats, we found differences in the expression of Th2-dependent cytokines IL-4 and IL-10. In addition, probiotics-treated CS rats showed lower levels of Th2 cytokines than CSO rats, suggesting that probiotics have the potential to modulate Th2 cytokines. However, until recently, few clinical studies and animal models have examined the relationship between specific bacteria and immune cytokines. To establish a clear association between specific bacterial taxa and allergy development, we used correlation analysis to investigate the potential link between bacteria and Th2 cytokines. *Ruminococcus*, *norank_f: Muribaculaceae*, and *unclassified_f:Prevotellaceae* were found to be positively associated with Th2 cytokines in our food allergy model, and *Ruminococcus* has been previously reported to be more prevalent in children with food sensitization (13). The relative abundance of these three bacteria was greater in CS_OVA group compared to VD_OVA group. Despite the lack of a significant difference in *unclassified_f:Prevotellaceae*, the probiotics-treated CS group had a substantially lower abundance of *Ruminococcus* and *norank_f: Muribaculaceae* than the CSO group following OVA sensitization. This shows that probiotic intervention can partially restore the microbiota of CS-delivered rats.

Several studies have suggested that food allergies cause damage to the gut barrier permeability (54, 55). Although some evidence shows that food allergy can reduce tight junction protein expression (42, 56), only CS-delivered sensitized rats exhibited lower expression of tight junction proteins than non-sensitized rats in our study. However, there was no such effect in VD rats. This might be explained by different food allergens or examination methods (e.g., q-RT-PCR, IHC, western blotting). Similarly, we also detected a significant difference in chao index, ace index, and PCoA between the sensitized and control groups in CS rats, but no comparable change was observed for the VD rats. Supplementation with *Lactobacillus* and *Bifidobacterium* elevated the expression level of tight junction proteins in sensitized CS rats. Thus, we conclude that healthy gut microbiota has the ability to protect the intestinal barrier against the dietary antigens and that altered intestinal barrier function is associated with a stronger anaphylactic response, which is consistent with the more severe clinical manifestations and higher levels of antibodies that were observed in our study. Together, we elucidate several pathways through which probiotics affect the development of food allergy, including their ability to modulate microbiota composition by suppressing certain harmful bacteria, regulate Th2 cytokines, and reverse impaired tight junction proteins. In addition, experimental and clinical data support a role for Th2 response in the regulation of intestinal barrier function, which is associated with an increase in intestinal permeability (57, 58). Further studies are needed to explore how Th2 cells modulate the tight junction proteins.

In summary, our study demonstrates that the intestinal microbiota plays an important role in the food allergy model of CS rats, and probiotic intervention is an effective strategy for the prevention of food allergy in this model. It is worth noting that the current overuse of CS delivery has resulted in alterations in microbiota and immunological development, and certain keystone species are undeniably crucial throughout critical stages of development (59). Vaginal seeding, which uses vaginal fluid to colonize the newborn gut with mother vaginal germs (30), is now deemed unsafe owing to the risk of harmful bacteria being transferred to the newborns (60). Dietary intervention represents a safer approach to vaginal seeding. Our findings suggest the possibility of gut microbiota-targeted therapies when CS delivery is an inevitable measure. Inconsistent with our choice, some studies support a reduced *Bacteroides* colonization pattern in early infancy (61, 62). Consequently, more clinical research is needed to see if probiotics can lower the risk of food allergy in children delivered *via* CS.

Our study had certain limitations. We compared vaginally delivered pups reared by birth mothers to CS-delivered pups reared by foster mothers. Instead of adding a cross-fostered group, we utilized vaginally born rats maintained by their biological moms as a control group because cross-fostering within the same strain has been shown to have little impact on bacterial communities (Shannon index, Chao index, and the relative abundance of the most predominant bacterial genera) or relative mRNA expression of immunoregulatory cytokines (TNFα, IFNγ, IL-4, and IL-10) in the gastrointestinal tract of the offspring (63). Similarly, a non-cross-fostered control has been successfully implemented to investigate the deficits mediated by mode of delivery (11, 64). Thus, for ethical reasons, we did not include extra groups in the current study to minimize animal usage. In addition, probiotic supplementation does not fully correct the gut microbiota dysbiosis. This might be due to the species applied, the methods employed, and/or the time of intervention. The vaginal microbiota of the mother, which is dominated by *Lactobacillus*, *Prevotella*, or *Sneathia*, is complicated, and microbial transmission to the infant occurs through the skin, oral mucosa, and nasal cavity at birth (20). It is not surprising that oral inoculation with certain species has a limited effect. Furthermore, the optimal duration of probiotic supplementation to prevent microbiota disruption is yet to be determined. However, such interventions still alleviate the symptoms of food allergy, which indicates the potential of probiotic supplementation to avert long-term negative consequences associated with CS.

## Data Availability Statement

The datasets generated for this study can be found in the National Center for Biotechnology Information (NCBI) database with accession code PRJNA743755.

## Ethics Statement

The animal study was reviewed and approved by the Ethics Committee on Animal Experiment of Qilu Hospital of Shandong University.

## Author Contributions

B-YJ, Y-NX, Z-XZ, X-YL, BL, and S-CF performed all experiments. B-YJ, L-XL, ZL, YL, and R-CZ performed data analysis. B-YJ wrote the manuscript. Y-QL, S-YL, and B-YJ designed the overall project and revised the manuscript. All authors contributed to the article and approved the submitted version.

## Funding

This study was supported by the National Natural Science Foundation of China (81873550 and 82070552) and Shandong Provincial Key Research and Development Program (Major Scientific and Technological Innovation Project) (No. 2019JZZY011007). This study was also supported by the Taishan Scholars Program of Shandong Province and National Clinical Research Center for Digestive Diseases supporting technology project (2015BAI13B07).

## Conflict of Interest

The authors declare that the research was conducted in the absence of any commercial or financial relationships that could be construed as a potential conflict of interest.

## Publisher’s Note

All claims expressed in this article are solely those of the authors and do not necessarily represent those of their affiliated organizations, or those of the publisher, the editors and the reviewers. Any product that may be evaluated in this article, or claim that may be made by its manufacturer, is not guaranteed or endorsed by the publisher.
