# Supplementary material for: Probiotic Interventions Alleviate Food Allergy Symptoms Correlated With Cesarean Section: A Murine Model
Source: Front Immunol. 2021 Sep 28;12:741371. doi: 10.3389/fimmu.2021.741371 (PMC8505808; doi:10.3389/fimmu.2021.741371)
Supplement: Supplementary file 1 [file DataSheet_1.pdf]

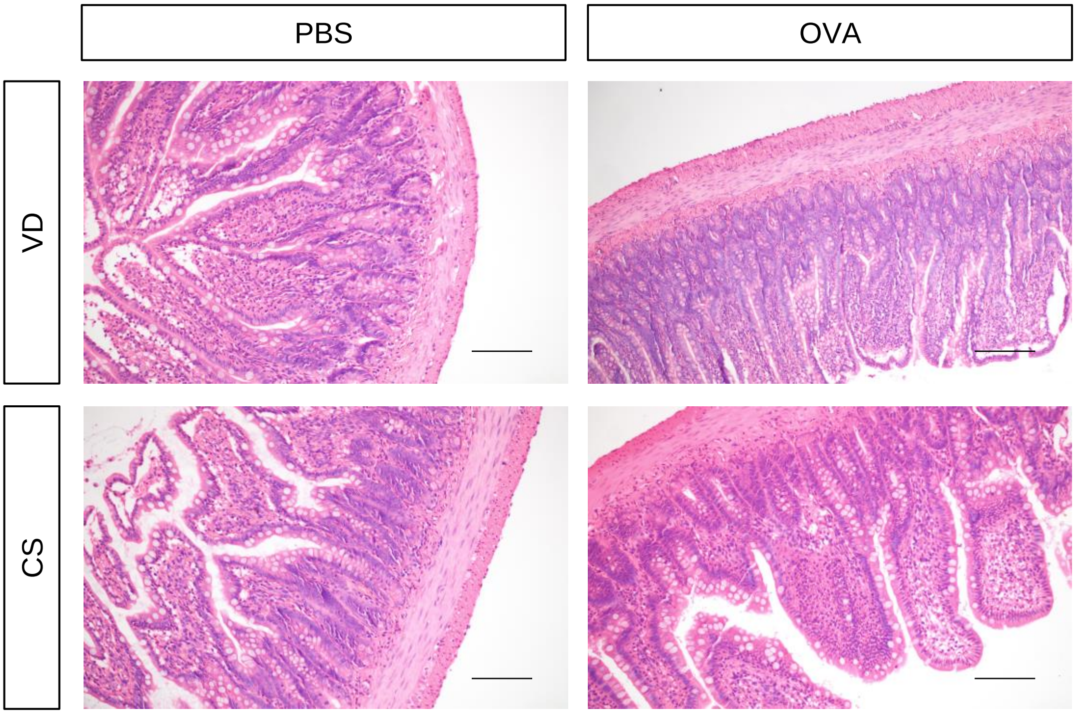

Supplemented Data Fig. S1 Sensitization of VD and CS rats with OVA does not result in intestinal pathology. Representative images of histological samples from PBS or OVA-treated rats delivered by CS or VD. All sections stained with hematoxylin and eosin. Scale bar = 100µm

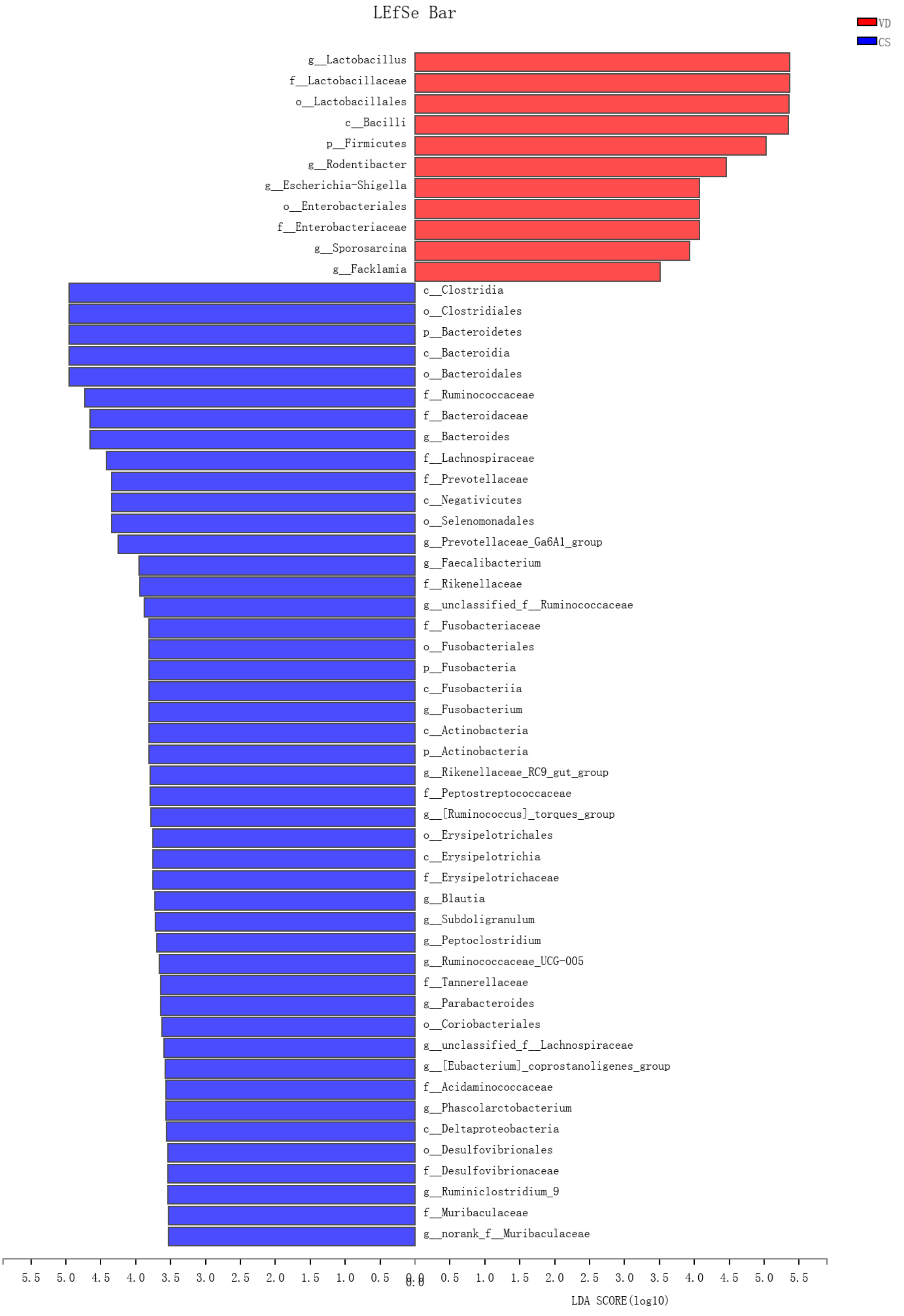

Supplemented Data Fig. S2 Mode of delivery affects the composition of the microbiota on Day 7.
LEfSe analysis of taxa that were significantly enriched in VD rats (red) or CS rats (blue) with the value of the Kruskal–Wallis rank-sum test set to 0.05 and an LDA cutoff value of 3.5

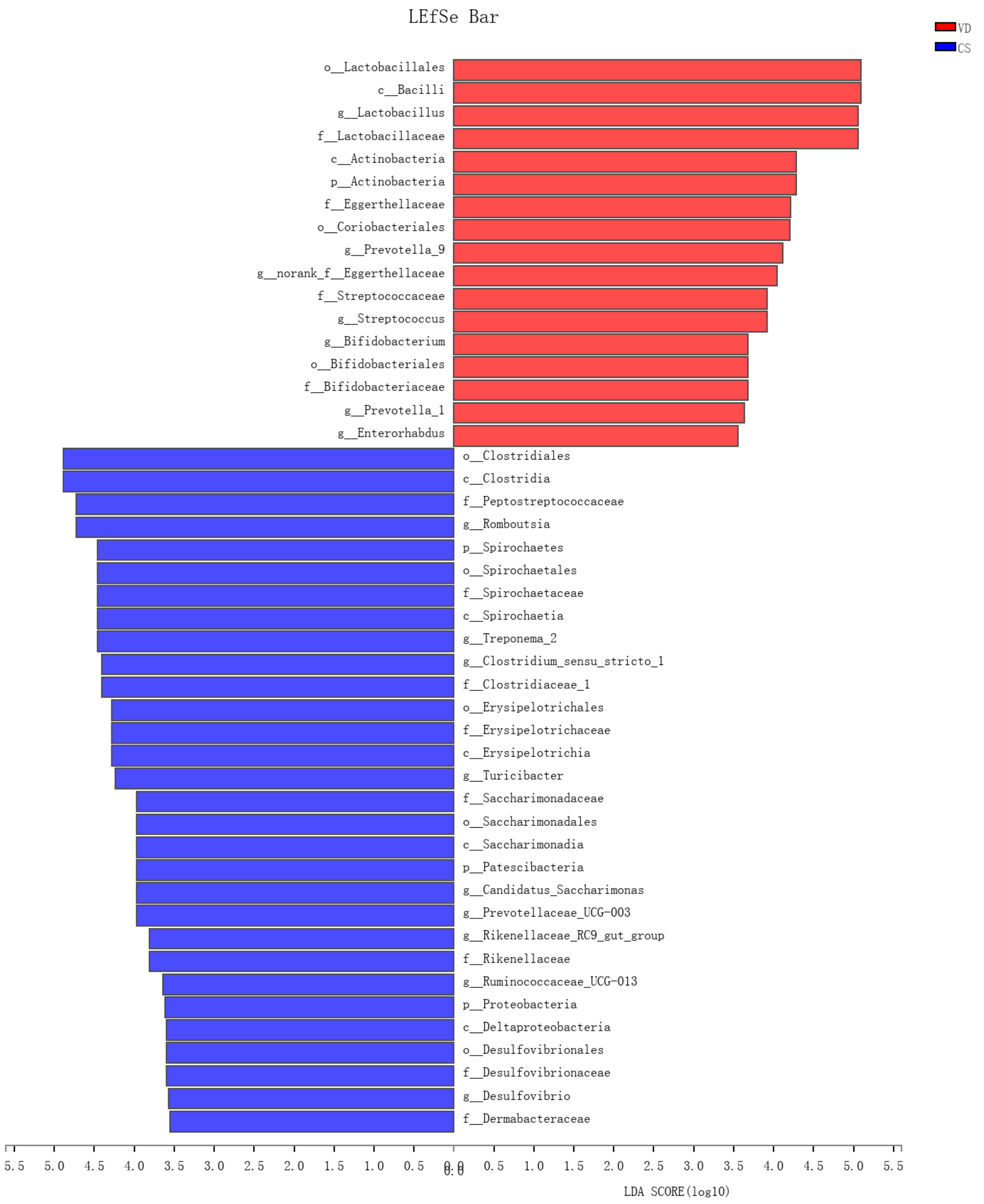

Supplemented Data Fig. S3 Mode of delivery affects the composition of the microbiota on Day 21.
LEfSe analysis of taxa that were significantly enriched in VD rats (red) or CS rats (blue) with the value of the Kruskal–Wallis rank-sum test set to 0.05 and an LDA cutoff value of 3.5
